# Editorial: Open issues in the diagnosis and management of tricuspid regurgitation

**DOI:** 10.3389/fcvm.2023.1189956

**Published:** 2023-04-17

**Authors:** Francesco Ancona, Antonio Mangieri, Marco Spartera, Maurizio Taramasso

**Affiliations:** ^1^ Cardiovascular Imaging Unit, Cardiothoracic Department, San Raffaele Scientific Institute, Milan, Italy; ^2^ Cardio Center, IRCCS Humanitas Research Hospital, Milan, Italy; ^3^ Cardiothoracic Department, Oxford University Hospitals NHS Foundation Trust, John Radcliffe Hospital, Oxford, United Kingdom; ^4^ Division of Cardiovascular Medicine, Radcliffe Department of Medicine, University of Oxford, OCMR, Oxford, United Kingdom; ^5^ HerzZentrum Hirslanden Zurich, Switzerland and University of Zurich, Switzerland

**Keywords:** tricuspid regurgitation, tricuspid valve, transcatheter tricuspid intervention, echocardiography, tricuspid valve imaging

**Editorial on the Research Topic** Open issues in the diagnosis and management of tricuspid regurgitation

Tricuspid valve regurgitation (TR) is a highly prevalent valvular heart disease with a prevalence of moderate or severe TR ranging from 7.1% in the Framingham Heart Study (1) to up to 16% in other cohorts (2).

Previously considered “the forgotten valve” perhaps because of lack of tailored treatment options as well as lack of data around its importance as a prognostic factor in cardiovascular disease, several data have shown that severe TR is an independent prognostic factor (3). This paved the ground for the development of treatment strategies aiming at reducing the degree of TR with a broader aim of improving cardiovascular outcomes as well as the quality of life in patients affected by this condition.

Conservative management of tricuspid regurgitation (TR) proved ineffective in reducing symptoms and progression of the right heart failure picture with worsening cardiac, renal, and liver functions. Recently, several percutaneous options have been trialed showing promising preliminary results in terms of improvement of symptoms and quality of life and reduction of hospitalization (4, 5).

**Figure 1:**
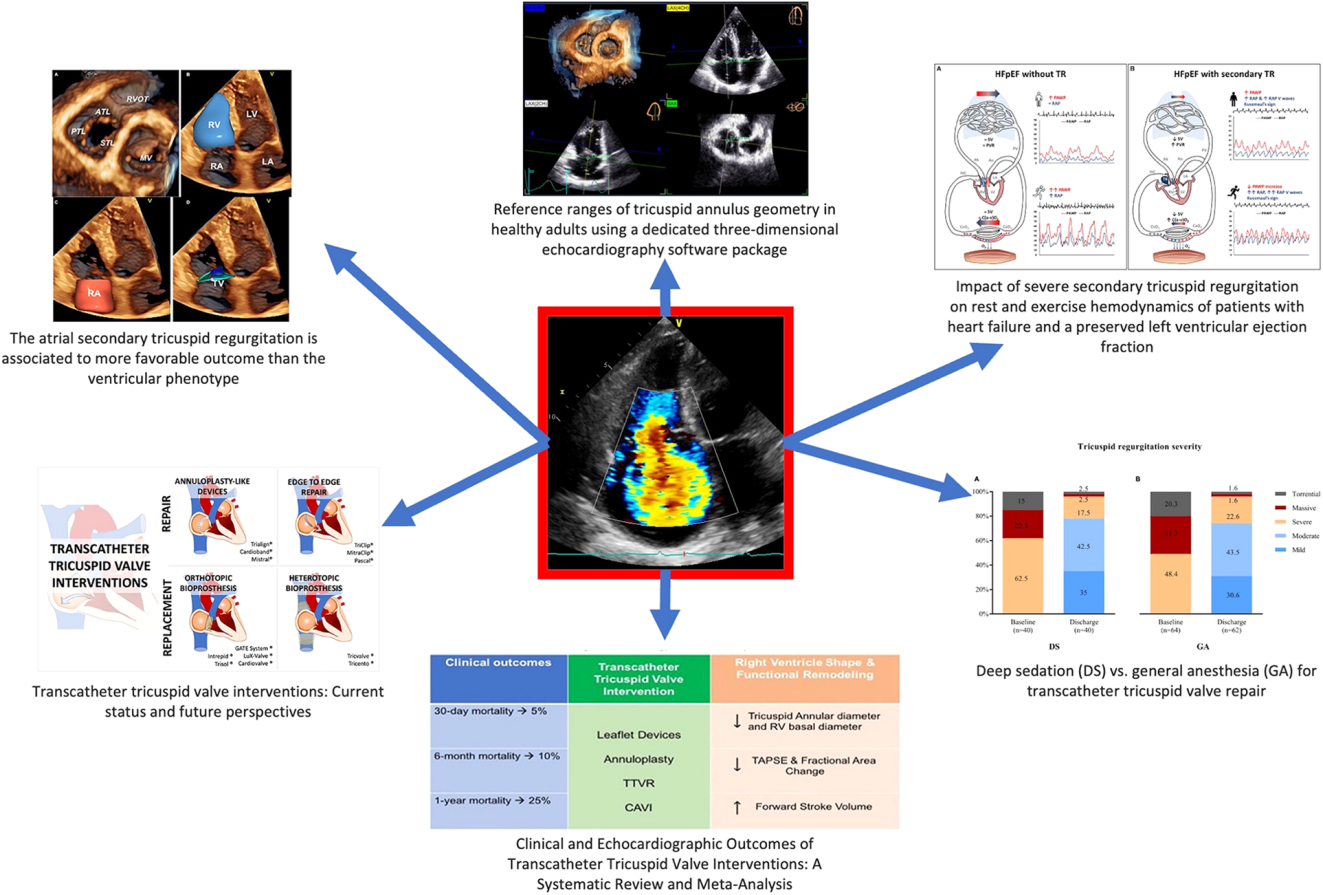
Open issues in the diagnosis and management of tricuspid regurgitation.

The aim of this special issue (Figure 1) is to give a focused update on the diagnostic aspects of TR, as well as appropriate management (both surgical and percutaneous) in different clinical setting.

The special issue will kick off with a discussion around the problem of quantification of TR and the evaluation of pathophysiological mechanisms. For instance, apart from the primary TR mechanisms (e.g., prolapse, flail, endocarditis, etc.), the more prevalent functional TR disease has been recently dissected into two different pathophysiological mechanisms: one due to annular remodeling (the so-called atrial TR) vs. one due to apical tethering forces (ventricular TR) (6). On this note, relevant data by Gavazzoni et al. highlighted the prognostic importance of the pathogenetic remodeling underlying TR, showing that atrial secondary TR is associated to more favorable outcome than the ventricular phenotype. Atrial secondary TR has shown less valvular remodeling and better RV longitudinal function, as evaluated with state-of-the-art methods. Both type of secondary TR and right ventricular function have an impact on mortality or event-free survival.

Further, Muraru et al. identified the reference values for tricuspid annulus geometry and dynamics using a commercially available three-dimensional echocardiography software package dedicated to the tricuspid valve, thus overcoming the intrinsic limitations of 2-dimensional linear measurements and giving sex-specific and indexed to BSA cut offs. Based on the wide scientific experience of their group on tricuspid valve and right heart chambers, this is a seminal work paving the way to subsequent possible prognostic relevance of 3-dimensional geometry of tricuspid annulus.

TR has an important additional impact of the hemodynamics of patient with heart failure with preserved ejection fraction (HFpEF), as highlighted by Baratto et al. The evaluation of cardio-pulmonary adaptation at rest and during exercise is an important aspect to be evaluated in patients with symptoms of heart failure. In particular severe functional TR complicating HFpEF led to pulmonary vascular de-recruitment and relative left heart underfilling, with important implications for HFpEF pathophysiology.

In the interventional part of this issue, Alperi et al. summed up the transcatheter systems used for TR correction under clinical use or clinical evaluation with their technical features with updated description of the current evidence in this challenging and evolving field.

Sannino et al. detailed the peculiar clinical and echocardiographic outcomes of transcatheter tricuspid valve interventions: in particular transcatheter options significantly reduces TR severity and increases forward stroke volume and is associated with improved survival at 1 year compared with patients without procedural success. It is important to underline that long-term outcomes compared with medical therapy await the results of ongoing pivotal trials, as the recent TRILUMINATE trial (5).

Haurand et al. studied if the use of deep sedation, as nowadays routinely used in transcatheter aortic valve implantation, impact on the procedural results of transcatheter TV repair: procedures performed under deep sedation, as opposed to the standard that is general anesthesia, were as effective and safe with similar low complication rates, showing the possible feasibility of less invasive anesthesiologic assistance during such procedures.

This special issue highlights the evolving field of TR interventions and the importance of diagnosis of degree of TR as well as the mechanisms underpinning such disease. The focus of such endeavor is relevant as it serves the higher aim of ameliorating the prognosis and symptoms of this large population of patients suffering from this no-more-forgotten condition.
